# Precision Medicine in Autoimmune Thyroiditis and Hypothyroidism

**DOI:** 10.3389/fphar.2021.750380

**Published:** 2021-11-17

**Authors:** Silvia Martina Ferrari, Francesca Ragusa, Giusy Elia, Sabrina Rosaria Paparo, Valeria Mazzi, Enke Baldini, Salvatore Benvenga, Alessandro Antonelli, Poupak Fallahi

**Affiliations:** ^1^ Department of Clinical and Experimental Medicine, University of Pisa, Pisa, Italy; ^2^ Department of Surgical, Medical and Molecular Pathology and Critical Area, University of Pisa, Pisa, Italy; ^3^ Department of Experimental Medicine, “Sapienza” University of Rome, Rome, Italy; ^4^ Department of Clinical and Experimental Medicine, University of Messina, Messina, Italy; ^5^ Master Program on Childhood, Adolescent and Women’s Endocrine Health, University of Messina, Messina, Italy; ^6^ Interdepartmental Program of Molecular and Clinical Endocrinology and Women’s Endocrine Health, Azienda Ospedaliera Universitaria Policlinico “G. Martino”, I-98125, Messina, Italy; ^7^ Department of Translational Research and New Technologies in Medicine and Surgery, University of Pisa, Pisa, Italy

**Keywords:** Hashimoto’s thyroiditis, autoimmune thyroid disorders, autoimmune thyroiditis, hypothyroidism, levothyroxine, thyroidectomy

## Abstract

Autoimmune thyroid diseases (AITD) are T-cell-mediated organ specific autoimmune disorders, deriving from an altered response of the immune system that leads to the immune attack to the thyroid. Hashimoto’s thyroiditis (HT) and Graves’ disease (GD) are the two principal AITD clinical presentations. Hypothyroidism and thyrotoxicosis are, respectively, the clinical hallmarks of HT and GD. Patients with autoimmune thyroiditis are treated daily with synthetic L-thyroxine (L-T4) at the dose of 1.5–1.7 μg/kg. Various L-T4 formulations are commercially available (tablet, liquid solution, or soft gel capsule). L-T4 in tablets is generally prescribed to treat hypothyroidism, whereas the liquid formulation, or soft gel capsules, can be administered in hypothyroid patients in case of malabsorption or in patients in therapy with drugs interfering with L-T4 absorption. Furthermore, myoinositol has a crucial role in thyroid autoimmunity and function. Clinical studies reported a significant decline in TSH and antithyroid autoantibodies levels after treatment with myoinositol + selenium in patients with subclinical hypothyroidism and autoimmune thyroiditis. Moreover, thyroidectomy can be rarely recommended in patients with autoimmune thyroiditis, with cosmetic reasons for a goiter, or with important signs or symptoms of local compression, or nodular disease with a “suspicious” cytology for malignancy. Furthermore, a recent randomized trial suggested that total thyroidectomy can improve quality of life and fatigue, while medical therapy did not. In this review, we overview currently available evidence in personalized medicine in patients with autoimmune thyroiditis and hypothyroidism. Further research is needed in larger population to investigate the effect of these new treatments on quality of life.

## Introduction

Autoimmune thyroiditis (AT) and Graves’ disease (GD) are the main autoimmune thyroid disorders (AITD), which are the most common autoimmune disorders (Romagnani, 1998; Antonelli et al., 2015).

AITD are distinguished by the breakdown of tolerance of the immune system against the thyroid (Antonelli et al., 2015). GD and AT are clinically characterized by thyrotoxicosis and hypothyroidism, respectively, by infiltrative autoreactive lymphocytes in the gland and the presence of serum antithyroid autoantibodies (ATA; Antonelli et al., 2015).

In the general population, women have a higher risk to develop AITD than men (∼4–8/1); the prevalence of AITD changes geographically, and it is more elevated in areas with iodine sufficiency (Antonelli et al., 2015). In the presence of AT, the frequency of hypothyroidism increases with age, such as the frequency of ATA (Antonelli et al., 2015).

Hashimoto’s thyroiditis (HT) leads to a chronic inflammation of the thyroidal tissue (McLeod and Cooper, 2012; Antonelli et al., 2015), wherein hypothyroidism is present in ∼25% of patients (McLeod and Cooper, 2012; Caturegli et al., 2014; Antonelli et al., 2015; Ragusa et al., 2019), and it was first described with significant signs of AT, lymphocytic infiltration, atrophy of follicular cells, fibrosis, and goiter. Thyroid hormones (TH) affect different organs and tissues, and for this reason, the symptoms and signs of hypothyroidism are various and unspecific, and can influence different systems (i.e., pulmonary, cardiovascular, hematopoietic, urinary, gastrointestinal, and reproductive; Galetta et al., 2008; Iddah and Macharia, 2013; Caturegli et al., 2014; Ragusa et al., 2019).

AITD are generally associated with another autoimmune disease [i.e., Sjogren syndrome (SS), systemic lupus erythematosus (SLE), sarcoidosis, systemic sclerosis (SSc), vitiligo, rheumatoid arthritis (RA), type 1 diabetes mellitus (T1D), celiac disease (CD), autoimmune gastritis, and HCV-related cryoglobulinemia], as reported in ∼19% of 3,069 patients with AT vs 2 gender- and age-matched control groups of 1,023 healthy subjects and 1,023 non-toxic multinodular goiter (MNG) subjects (Fallahi et al., 2016a). Moreover, the association of three different autoimmune diseases was reported in AT with respect to controls (Fallahi et al., 2016a). Another study reported similar data in 3,209 GD subjects (984 with Graves’ ophthalmopathy) vs 1,069 controls, 1,069 MNG, and 1,069 AT patients, showing the prevalence of another autoimmune disease in ∼17% of GD subjects (Ferrari et al., 2019a).

Furthermore, epidemiological studies have reported that AITD can be associated with papillary thyroid cancer (PTC). Elevated TSH levels were associated with the PTC risk in 13,738 AT patients (Fiore et al., 2011). Conversely, other papers demonstrated that both thyroid autoimmunity and high TSH levels are independent risk factors for PTC (Boi et al., 2013).

The association between inflammation and TC involves different components of the immune system (macrophages, lymphocytes, cytokines, and chemokines) (Antonelli et al., 1999; Ferrari et al., 2019b; Ferrari et al., 2019c).

## Genetic susceptibility And Environmental Factors

### Genetic Susceptibility

Different observations are at the basis of the genetic susceptibility to AITD: (1) the familial clustering (25% of AITD in siblings of AITD subjects); (2) AITD sibling risk ratio of ∼17; and (3) a strong prevalence of ATA in siblings of AITD patients (Brix and Hegedüs, 2012).

The association among AITD, the presence of ATA and certain genes [i.e., human leukocyte antigen (HLA), IL2RA, CTLA4, and PTPN22], has been demonstrated, and also other AITD genes were identified by genome-wide association studies (GWAS) [i.e., HLA class I, and TSH receptor (TSH-R)] (Simmonds, 2013; Tomer and Davies, 2013). Other AITD risk genes have been identified by GWAS and Immunochip techniques (i.e., FOXE1, BACH2, RNASET2 and GDCG4p14; Simmonds, 2013).

Of particular interest, 7/11 known susceptibility genes are involved in the role of T cells, suggesting their importance in the immune-pathogenesis of AITD (Antonelli et al., 2015), even if chronic AT can occur in the absence of serum ATA (Rotondi et al., 2014a). A case-control retrospective study enrolled 55 patients with serum negative chronic AT and 110 patients with chronic AT. Patients with serum negative chronic AT had significantly lower mean TSH levels, higher mean FT4 levels, comparable FT3 levels, and a significantly lower mean thyroid volume vs patients with chronic AT. The data suggested that patients with serum negative chronic AT would display a milder clinical phenotype (Rotondi et al., 2014b).

Furthermore, a monogenic form of AITD was first reported in a family with autosomal dominant inheritance of HT (Lo et al., 2018).

### Environmental Factors

In iodine sufficient areas, hypothyroidism is due mainly to HT. A lower AITD prevalence is shown in iodine-deficient areas, whereas an exaggerated iodine intake is associated with a higher AITD prevalence (Iddah and Macharia, 2013; Ferrari et al., 2017a).

Smoking habits are a risk factor for Graves’ hyperthyroidism (Perricone et al., 2016); whereas, overall, Graves’ ophthalmopathy reduce the risk of hypothyroidism (Carlé et al., 2012).

Radiation exposure is another risk factor, and elevated ATA levels have been reported in children after nuclear disasters, with a subsequent raised risk of TC and thyroid dysfunctions (Antonelli et al., 1996).

The role of viruses in the pathogenesis of AITD has been evaluated with contrasting findings; however, an association between AITD and hepatitis C virus chronic infection (HCV) has been confirmed (Menconi et al., 2011; Ferrari et al., 2013; Ferri et al., 2015; Zignego et al., 2015; Ferrari et al., 2020).

Also some drugs (i.e., tyrosine kinase inhibitors) are an emerging cause of primary hypothyroidism (Fallahi et al., 2014).

Selenium and vitamin D deficiency is a possible AITD risk factor, too. The thyroid expresses specific selenoproteins, and a decreased selenium assumption is an AITD risk factor (Duntas, 2010; Wang et al., 2018; Benvenga et al., 2020a).

Another AITD risk factor is stress, both emotional and psychological, that perhaps owing to the effect of cortisol on immune cells, followed by immune hyperactivity, can lead to thyroid autoimmunity (Iddah and Macharia, 2013).

## Levothyroxine Treatment

The synthetic hormone L-T4 is recommended as therapy of hypothyroidism-related conditions as it has a chemical structure similar to T4 (Miccoli et al., 1993; Fallahi et al., 2017a).

The tablet formulation of L-T4 contains the stable salt sodium L-T4, along with various excipients, and it needs an acid gastric pH to be dissolved (Centanni et al., 2006; Virili et al., 2019a). In the absence of factors that alter L-T4 absorption, ∼70% of tablet L-T4 is absorbed into the duodenum and jejunum (Fallahi et al., 2017a).

Thanks to more sensitive TSH assays, nowadays, 1.5–1.7 μg/kg body weight is the ideal daily L-T4 replacement dose, able to obtain normal TSH levels in most hypothyroid subjects (Caturegli et al., 2014). In particular, in patients with serum negative chronic AT, the required dose of L-T4 appears to be lower, as reported by a study conducted in 49 hypothyroid patients with serum negative chronic AT and in 98 hypothyroid patients with HT (Croce et al., 2020).

In spite of this, owing to different interfering issues (Miccoli et al., 1993), ∼20–50% of patients do not respond to the L-T4 treatment (Eligar et al., 2016; Virili et al., 2019b) and require a higher dose and monitoring (Ernst et al., 2017). Once excluded, a possible pseudomalabsorption linked to a scarce compliance with the prescribed regimen, gastrointestinal disorders, or interfering drugs can cause a decrease in intestinal absorption of L-T4 and are considered the main cause of refractory hypothyroidism (Virili et al., 2019b).

### Levothyroxine Tablets Malabsorption

L-T4 tablets are usually taken before breakfast. It has been shown that the assumption of L-T4 10 min before coffee in the morning reduces its absorption (Benvenga et al., 2008). A prospective, open-label, randomized, cross-over study compared the L-T4 administration during fasting with that during breakfast. In patients receiving L-T4 during breakfast, TSH was more elevated than in those during fasting (2.89 vs 1.9 mIU/L), suggesting that it is better to take L-T4 during a fasting state (Perez et al., 2013).

Moreover, different intestinal or gastric diseases can alter the L-T4 tablet absorption (Formenti et al., 2015). For this reason, in patients affected by *Helicobacter pylori* (HP) gastritis, or atrophic gastritis, who have an altered acid secretion, the daily L-T4 requirement is raised by 22–34% (Annibale et al., 1997; Yao and Forte, 2003; Centanni et al., 2006).

Proton-pump inhibitors (PPI) can reduce L-T4 absorption, too. The effect of PPI on serum TSH in 37 euthyroid subjects who had been administered with stable L-T4 for at least 6 months was evaluated. From before the PPI treatment to 2 months after it, the mean change in TSH was higher than in controls. The data indicated that in hypothyroid patients treated with L-T4 and PPI, further TH measurement and/or adjustment of the L-T4 dose might be necessary (Sachmechi et al., 2007).

Intestinal disorders can lead to an increased need for L-T4, too (Rostom et al., 2006). The association of different autoimmune disorders is well-known (Galetta et al., 2008; Formenti et al., 2015; Fallahi et al., 2016a), and CD and AITD are cognate disorders (Fallahi et al., 2019).

The L-T4 dose in 35 hypothyroid patients with atypical CD and AITD has been evaluated vs patients with AITD only. The dosage should be increased up to 50% if CD patients did not follow a strict gluten-free diet (Virili et al., 2012).

Lactose intolerance (LI) can lead to a reduction of L-T4 absorption (Asik et al., 2014). The L-T4 dose to normalize TSH was evaluated in 34 AITD hypothyroid patients with LI who did not follow a lactose-free diet. Moreover, in another study in LI AITD patients, the dose of L-T4 to normalize TSH was 1.81 μg/kg/day while it was of 1.31 μg/kg/day in AITD patients without LI (Cellini et al., 2014).

Also bariatric surgery can cause reduction of L-T4 absorption (Pirola et al., 2013) as restrictive surgical procedures that increase gastric pH can alter drug dissolution and solubility (Padwal et al., 2010).

### Novel Oral Levothyroxine Formulations

Refractory hypothyroidism and the need to increase the “normal dose” of L-T4 (Centanni et al., 2017; Fallahi et al., 2021) has led to the development of new L-T4 preparations, soft gel capsule and the liquid formulation.

#### Oral Liquid Levothyroxine

The liquid preparation is bioequivalent with the tablet L-T4 (Yue et al., 2012). It contains L-T4, ethanol, and glycerin, and it does not need an acid gastric pH to be dissolved (Vita et al., 2014a).

Two meta-analyses evaluated the efficacy of the liquid formulation: the first one suggested that patients treated with L-T4 tablets with suboptimal TSH levels can obtain the expected TSH upon the switch to liquid L-T4 with the same dose (Virili et al., 2018) and the second one reported that the liquid L-T4 was more effective (in comparison to tablets) in subjects in presence/absence of reduced absorption both in replacement such as in suppressive treatment (Laurent et al., 2018).

Liquid L-T4 has been considered also in 78 newborns with congenital hypothyroidism, reporting a TSH inhibition rate that could be associated with a stronger absorption (compared to tablets; Peroni et al., 2014). Another study confirmed these data (Pirola et al., 2014).

Furthermore, the oral liquid L-T4 can maintain better circulation of normal TSH with respect to tablets also in elderly (Cappelli et al., 2014) and during pregnancy (Cappelli et al., 2015).

#### Soft Gel Capsule Preparation

In soft gel capsule, sodium L-T4 is in glycerin and water, and maintained into a gelatin matrix. It is free of gluten, lactose, alcohol, dyes, or sugar (Vita et al., 2014a). It is rapidly dissolved in the acid gastric pH.

Another study investigated is if soft gel capsule preparation can bypass the reduced absorption associated with the ingestion of coffee in eight patients, who were switched from the tablets to the capsule for 6 months with the same L-T4 dosage (Vita et al., 2013). Patients followed a proper habit on days 1–90, taking coffee 1 h after the drug assumption, while they followed an improper habit on days 91–180 taking coffee ≤5 min after the capsule. The obtained data suggested that soft gel capsule is effective in subjects with an improper habit of taking L-T4 (Vita et al., 2013).

Another study evaluated the daily required L-T4 dose in 103 thyroidectomized patients. Even if the L-T4 requirement to attain optimal TSH levels was similar among patients receiving soft gel capsules and tablets, mean TSH decreased by 28% in those receiving the soft gel vs tablets (Di Donna et al., 2018).

Moreover, the effect of the switch from tablets to soft gel capsule preparation was investigated in hypothyroid patients, without increasing the L-T4 dose. In 11/18 patients treated with L-T4 tablets, serum TSH was normal and upon the switch in 16/18 (with a lower median TSH value; Trimboli et al., 2018).

### Advances in the Treatment of Hypothyroidism with the New Levothyroxine Preparations

The recently marketed novel preparations of L-T4 have led to a significant reduction in TSH variability in hypothyroid patients, in comparison to tablets.

Novel L-T4 preparations can be administered in hypothyroid patients in case of malabsorption:

- with food and beverages interference, or in subjects who do not wish to ingest L-T4 30–60 min before breakfast (Cappelli et al., 2016; Guglielmi et al., 2018);

- deriving from an increased gastric pH (Centanni et al., 2006; Lahner et al., 2009; Vita et al., 2014b; Lahner et al., 2014; Fallahi et al., 2016b; Cellini et al., 2017; Ribichini et al., 2017; Guzman-Prado et al., 2020);

- following bariatric surgery or with intestinal malabsorption (Pirola et al., 2013; Fallahi et al., 2017b);

- induced by interferent drugs (Fallahi et al., 2014);

- in case of typical or atypical CD (Virili et al., 2012; Zubarik et al., 2015; De Carvalho et al., 2018), or LI (Asik et al., 2014; Fallahi et al., 2017c; Fallahi et al., 2019);

- who can not swallow the tablets (Pirola et al., 2014).

Moreover, both in patients with malabsorptive issues or with no malabsorption, the oral liquid L-T4 is able to maintain more efficiently, than L-T4 tablets, normal TSH values in hypothyroid patients in the long-term follow-up (Antonelli et al., 2021).

## Myoinositol and Selenium in Patients with Autoimmune Thyroiditis and Hypothyroidism

Inositol is a compound soluble in water (Benvenga et al., 2020a), whose most abundant form is myoinositol (Benvenga and Antonelli, 2016).

Myoinositol is the precursor of phosphoinositides, and it takes part into various cellular processes (Fallahi et al., 2018).

In humans, the raised values of TSH were reduced in AT patients with subclinical hypothyroidism after therapy with myoinositol and seleno-methionine, such as AbTg and AbTPO levels. Seleno-methionine alone did not induce the same decrease (Nordio and Pajalich, 2013).

Selenium is an essential micronutrient which is necessary for cellular function, and it exercises its function in the form of the amino acid selenocysteine within selenoproteins (Duntas and Benvenga, 2015).

It is well-known that selenium is determinant in thyroid autoimmunity (Antonelli et al., 2015). Selenium deficiency is associated with an elevated prevalence of AT, owing to a reduced activity of selenium-dependent enzymes within thyrocytes and the immune system (Mazokopakis et al., 2007; Duntas and Benvenga, 2015).

Considering the pathogenetical link between AITD and environmental factors that can trigger oxidative stress and the antioxidant property of selenium, the possible supplementation with sodium selenite or seleno-methionine has been evaluated in AITD (Duntas and Benvenga, 2015). A favorable effect of the combination of myoinositol and seleno-methionine has been shown in patients with subclinical hypothyroidism (Nordio and Pajalich, 2013; Benvenga et al., 2020b).

The immune-modulating action of myoinositol combined with seleno-methionine was investigated in 21 euthyroid AT patients, who received myoinositol in combination with selenium (600 mg/83 μg) tablets, for 6 months twice a day (Ferrari et al., 2017b). After the treatment, TSH levels significantly declined, such as ATA, in particular in AT patients with TSH in the high normal range. These data suggested that myoinositol and seleno-methionine in combination can decrease the risk of a worsening of hypothyroidism. Moreover, after the treatment, also serum CXCL10 chemokine levels declined with respect to basal values, confirming the immune-modulatory effect of combined myoinositol and selenium (Ferrari et al., 2017b).

CXCL10 (or IP-10, the IFN-γ-inducible protein 10) is an IFN-γ-inducible chemokine that is implicated in lymphocyte infiltration and thyroid destruction in HT (Antonelli et al., 2011; Fallahi et al., 2020). The immune-modulatory effect of the combination of myoinositol and selenium on CXCL10 secretion suggests it could reduce the Th1 immune response (Cantrell, 2015).

Another paper evaluated the effect of different additions of myoinositol, seleno-methionine, or their combination on *in vitro* peripheral blood mononuclear cells (PBMC) obtained from three controls and eight HT women, treated with hydrogen peroxide (H2O2; Benvenga et al., 2017). H2O2 alone reduced dose-dependently PBMC proliferation in either groups, and cell vitality by 5% in control subjects and 10% in HT patients, but vitality was rescued by the following additions, inhibiting also the genotoxic effect. Chemokines increased after H2O2 alone, while the following additions dose-dependently diminished these levels, especially with myoinositol + seleno-methionine (Benvenga et al., 2017). Moreover, H2O2 increased the apoptosis in primary thyrocytes and decreased the proliferation, reducing also IFN-γ-induced CXCL10 secretion. The combination of myoinositol + seleno-methionine reduced the cytokine-induced secretion of CXCL10 both in presence/absence of H2O2, while seleno-methionine alone had no significant effect. This suggested a protective effect of myoinositol in thyrocytes (Ferrari et al., 2018).

An observational and retrospective study evaluated TH (after 6 and 12 months of treatment) in HT patients (both euthyroid, such as with subclinical hypothyroidism) and divided them in: untreated, treated with seleno-methionine alone (83 μg/day), and treated with seleno-methionine + myoinositol (83 μg/day + 600 mg/day). TSH levels were reduced (31–38%) in HT patients treated with seleno-methionine and/or seleno-methionine + myoinositol, while TSH increased in untreated patients. In particular, the TSH decrease was shown earlier in patients receiving seleno-methionine + myoinositol than in those treated with seleno-methionine alone (Pace et al., 2020).

Furthermore, the effect of myoinositol has been investigated in 86 patients with subclinical hypothyroidism and HT, who received 600 mg myoinositol and 83 μg seleno-methionine for 6 months. A significant amelioration in TSH values and in the quality of life of the patients was reported (Nordio and Basciani, 2017a). Another study was conducted in 168 patients with HT and TSH between 3 and 6 mIU/mL, who were subdivided into two groups: treated with myoinositol + seleno-methionine (600 mg + 83 μg, respectively) and treated with seleno-methionine (83 μg). TSH, FT4, AbTPO, and AbTg improved only in patients treated with myoinositol + seleno-methionine (Nordio and Basciani, 2017b).

A placebo-controlled randomized prospective study evaluated the short-term effect of L-seleno-methionine on the thyroid function in 76 euthyroid HT patients; among them, 38 received L-seleno-methionine (166 μg/die) and 38 received placebo for 6 months. TSH, FT4, FT3, AbTPO, thyroid echogenicity, and CXCL10 were not statistically different between the two groups of patients at time 0 after 3 and 6 months. The data suggested that the short-term L-seleno-methionine supplementation has a slight impact on the natural course in euthyroid HT (Esposito et al., 2017).

## The Role of Thyroidectomy in Autoimmune Thyroiditis and Hypothyroidism

Thyroidectomy can be rarely recommended in AT patients, with cosmetic reasons for a goiter, or with important signs or symptoms of local compression, or nodular disease with a “suspicious” cytology for malignancy.

Moreover, in some patients with HT, symptoms persist despite their euthyroid status while receiving hormone substitution. The total removal of the antigenic tissue through total thyroidectomy appears to attenuate the autoimmune response (Chiovato et al., 2003) and ameliorate symptoms (Promberger et al., 2014).

A randomized trial has been conducted (ClinicalTrials.gov: NCT02319538) in 150 patients (with an age of 18–79 years) with persistent HT-associated symptoms even if in euthyroidism while in treatment with L-T4 therapy and with serum AbTPO >1,000 IU/ml (Guldvog et al., 2019). In the follow-up, only the surgical group of patients had an improvement, with an increase in the mean health score from 38 to 64 points, at 18 months. Fatigue score and chronic fatigue frequency decreased. Median circulating AbTPO levels were reduced from 2,232 to 152 IU/mL. Total thyroidectomy ameliorated the quality of life and fatigue in these patients, but not the medical treatment (Guldvog et al., 2019).

## Conclusion

HT causes a chronic inflammation of the thyroid tissue (McLeod and Cooper, 2012; Antonelli et al., 2015), with a condition of hypothyroidism in ∼25% of patients (McLeod and Cooper, 2012; Caturegli et al., 2014; Antonelli et al., 2015; Ragusa et al., 2019), and it was first described with significant signs of AT, lymphocytic infiltration, atrophy of follicular cells, fibrosis, and goiter.

The synthetic hormone L-T4 is recommended as therapy of hypothyroidism-related conditions as it has a chemical structure similar to T4 (Fallahi et al., 2017a).

The tablet formulation of L-T4 needs an acid gastric pH for its absorption (Centanni et al., 2006). In the absence of factors that alter L-T4 absorption, ∼70% of tablet L-T4 is absorbed into the duodenum and jejunum (Fallahi et al., 2017a).

Thanks to more sensitive TSH assays, nowadays, the dose of 1.5–1.7 μg/kg body weight is the ideal daily L-T4 replacement dose, able to obtain normal TSH levels in most hypothyroid subjects (Caturegli et al., 2014).

In spite of this, owing to different interfering issues (Virili et al., 2019b), ∼20–50% of patients do not respond to the L-T4 treatment (Eligar et al., 2016; Virili et al., 2019b) and require a higher dose (Ernst et al., 2017). Once excluded, a possible pseudomalabsorption linked to a scarce compliance with the prescribed regimen, gastrointestinal disorders, or interfering drugs can cause an altered intestinal absorption of L-T4 and can lead to refractory hypothyroidism (Virili et al., 2019b).

Refractory hypothyroidism and the need to increase the “normal dose” of L-T4 (Centanni et al., 2017; Fallahi et al., 2021) has led to the development of new L-T4 formulations, the oral liquid preparation and soft gel capsule, that have permitted a significant reduction in TSH variability in hypothyroidism, in comparison to tablets.

Moreover, myoinositol has a key role in thyroid autoimmunity and function. A significant decline in TSH and ATA levels has been reported in patients with subclinical hypothyroidism and AT after treatment with myoinositol + selenium, corroborating the immune-modulatory effect of myoinositol (Ferrari et al., 2017b).

Furthermore, thyroidectomy can be rarely recommended in patients with AT. A recent randomized trial suggested that total thyroidectomy can ameliorate quality of life and fatigue in these patients (Guldvog et al., 2019).

In conclusion, in this novel era of precision medicine, further research is needed in larger population to investigate the effect of these new treatments on quality of life.
